# In Search of Autonomy: Dancing With Rules

**DOI:** 10.3389/fpsyg.2021.717590

**Published:** 2021-12-09

**Authors:** Frode Heldal, Erlend Dehlin

**Affiliations:** ^1^NTNU Business School, Norwegian University of Science and Technology, Trondheim, Norway; ^2^Department of Teacher Education, Norwegian University of Science and Technology, Trondheim, Norway

**Keywords:** practical judgement, autonomy, interactionist perspective, rule breaking, management

## Abstract

**Purpose:** Autonomy in organisations cannot exist without rules nor relationships. Yet, previous research tends to elicit understandings of autonomy as freedom from external constraints to enact free individual will. And there are numerous positive effects related to autonomy at work. But research has not kept pace with modern-day organisations that are highly flexible and dynamic. Current understandings of autonomy are static. Autonomy is mainly regarded as something individuals possess, more or less constricted by rules. Our purpose is to contribute a more flexible and practice-oriented concept of autonomy to answer the research question: How is autonomy developed and practiced in relation to formal rules in high-risk organisations?

**Design:** To investigate autonomy as a dynamic and flexible concept, we draw on two case studies comprised of a total of 52 interviews and more than 10 h of observation. The cases include a factory and a hospital unit.

**Findings:** We suggest, based on the data, that autonomy is a relational phenomenon. We suggest four different autonomy-rule dynamics: Passive, loyal, self-promoting, and co-generative learning.

**Research Implications:** Regarding autonomy as relational rather than individual contributes to our understanding of organisations as always in the making. In this, we emphasise the interactive element of autonomy.

**Practical Implications:** Practitioners and managers may use our suggestions to work with autonomy in a different way, spurring creativity and improvisation by constructively using rules.

**Originality:** Little research has paid attention to the concept of autonomy (despite its importance), and arguably, a trend in the available research concerns a commodification of the phenomenon, primarily aligning autonomy with (degrees of) negative freedom and individual decision making. We unpack the concept with attention to interaction – what we have called dancing with rules.

## Introduction: In Search of Autonomy

For more than half a century research has extensively documented the benefits of job autonomy for employee well-being, motivation, and productivity (French et al., 1960; Giacolone and Greenberg, 1997). Even though job autonomy is hailed as important, research has yet to agree on a common understanding of the concept, resulting in a variety of, and to some extent conflicting, denotations. Whereas some link job autonomy to positive freedom (Taylor, 1985) underscoring the individual freedom *to* be innovative and make choices (Hackman and Oldman, 1976), others emphasise negative freedom (Taylor, 1985) as represented in different notions of being free *from* external imposition. Examples of the latter include concepts like independence, sovereignty, and self-governance (Wiedner and Mantere, 2019).

This manuscript recognises and addresses the complexity of autonomy as a workplace phenomenon, and seeks to offer a broad conceptual understanding on the basis of process thinking (Mead, 1934; Weick, 1995; Chia, 2002). With a particular eye on social dialectics and (organisational) context, the manuscript presents empirical findings that indicate intricate and vital connections between job autonomy and organisational rules. Contrary to a (polarised) perspective on autonomy as merely an individual attribute, either pertaining to positive or negative freedom, ideas are advanced that job autonomy and rules form an internal and intricate relationship, and that autonomy in work practice is about interweaving individual freedom with organisational rules and social structures. Positive freedom, the manuscript argues, comes about by way of negative relations to the social and organisational sphere, and as the rule becomes the instrument for both individual and organisational development, job autonomy is more about dialectic growth than individual attribution.

As the manuscript aims to advance a fuller understanding of both the significance and complexity of job autonomy as related to organisational rules, we start by providing a theory section offering a review of prevalent literature. Here we have a particular focus on conceptual shortcomings and inconsistencies in need of resolving, for the purpose of advancing a rich, nuanced, and practically relevant understanding of the complex and turbulent nature of autonomy and rules in contemporary organisational life. As the empirical basis for the following discussion, we present data from two high-risk organisations. High-risk organisations are contexts in which rules may be more prominent than in other organisations, but yet also contexts in which autonomy is hailed as important (Antonsen et al., 2017). As the guiding research question, we ask: How is autonomy developed and practiced in relation to formal rules in high-risk organisations?

## Theory

Research concurs on the positive benefits of autonomy at work, for instance, that it is positively related to job satisfaction, motivation, and job performance (e.g., Saragih, 2011; Kubicek et al., 2017). Autonomy is further seen as a precursor for “doing good,” as in the concept of pro-social rule-breaking (Morrison, 2006). Linguistic clarity or agreement as to what constitutes “job autonomy” in an organisational research frame is, however, hard to pin down, and though (for most) shares a foundation in individual freedom, some conceptual takes draw on notions of positive freedom, individual choice, and creativity, whereas others on notions of negative freedom; to be free from external imposition/coercion/direction. We will deal with each successively, and conscious of the pitfalls of conceptual dualism and polarisation, we will construe a line of reasoning in which job autonomy is first and foremost a process of growth, becoming, and positive freedom, and where rules not only constraint negative freedom, but provide the necessary instruments for individual and organisational development (akin to a positive freedom-perspective). Autonomy involves, in this sense, an emergence of practical wisdom, a continuous offspring from a dialectic of negative and positive freedom (Dreyfus and Dreyfus, 1980).

### Autonomy at Work

Wiedner and Mantere (2019) argue that autonomy is seldom defined in the organisation and management literature, but that it is often associated with terms such as “independence,” “sovereignty,” and “self-governance.” Similarly, Pollard (2003) claims that autonomy is typically defined through concepts like “self-governance and “self-regulation,” along with the somewhat more vague “independence” and “freedom” (Pollard, 2003). Conceptual clarity, then, is left at providing some vague anchoring in individuality and personal privilege, largely associated with “negative freedom,” referring to the ability of an individual to act freely, without obstruction or interference from external powers (Taylor, 1985; Wiedner and Mantere, 2019). Consequently, autonomy is often contrasted with managerial control (Heldal, 2015). This is similar to a difference in autonomy often seen in professional organisations, where some distinguish between professional autonomy and clinical autonomy. Professional autonomy marks the parameters of clinical autonomy, while clinical autonomy may be understood as the framing of everyday work activities (Timmermans, 2005; Heldal, 2015). And connected to practical settings, the individual perspective is even more accentuated in motivational theories such as the self-determination theory, where autonomy is seen as an important predictor of intrinsic motivation (Kubicek et al., 2017).

“…having [our emphasis] more autonomy may mean qualitatively different things in work settings that are highly flexible and characterized by indirect control than in work settings that are highly regulated and externally controlled.” (Kubicek et al., 2017, 51)

Further, distinctions are commonly made between “work autonomy” (selecting work methods, also called “methods autonomy”), “working time autonomy” (the choosing of timing), and “workplace autonomy” (*where* autonomy is exercised), and lastly, “task autonomy” (*how* a task is carried out). While these aspects of job autonomy are situated at the task level of a job, recent developments such as the expansion of flexible work arrangements, have brought about a greater focus on autonomy at the job level: Employees now have autonomy in deciding when (working time autonomy) and where (workplace autonomy) they perform their jobs (Ala-Mursula et al., 2005; Nijp et al., 2012).

Further, autonomy is regularly associated with the exercise of individual choice, the power to make and act upon decisions (Henry and Fryer, 1995). Autonomy as a key ingredient in specific work practices that require varying degrees of competence follows in a similar vein (Breaugh, 1985). Kubicek et al. (2017, 46) states that autonomy is about how employees pursue their work tasks and that:

“…the regulation of work has to an increasing extent been handed over to individual workers, who are given greater autonomy in performing their jobs…”

Some sub-topics run through the research presented, the first of which is that autonomy constitutes a trend associated with de-regulation of the workplace. Second, autonomy is presented as some*thing* that can be given to individual workers, like a gift (Mintzberg, 1999). Notably, this simultaneously implies a commodification of autonomy as well as an attribution of the commodity to the individual worker – that some have more of it than others. Third, there is arguably a strong notion of negative freedom underpinning the literature, as autonomy follows a logic by which more individual freedom is achieved at the expense of less hierarchical control. That individual freedom is, however, not so much tied to personal growth and creative development (as a practitioner), but to perform tasks under less supervision. To the extent that positive freedom is involved, for instance as in making choices, these are decisions made on the ground of given parameters of control: Autonomy becomes an exercise of (limited) choice within a controlled system, and only to a marginal degree an open-ended process of growth and development. The next section elaborates these points further and discusses potential pitfalls for understanding autonomy at work and how to understand autonomy as a relationship with pro-active practitioners.

### Autonomy and Freedom

Positive freedom is commonly described as the liberty to act creatively out of the free will, whereas negative freedom denotes liberty from external restraint (Taylor, 1985). When autonomy becomes largely a measure of negative freedom and is operationalised through different categories at the task or job level, it facilitates the birth of concepts like “organisational autonomy” (Verhoest et al., 2004) and “job autonomy” (Hackman and Oldman, 1976). As an attribute of the system, autonomy becomes a commodity, a thing unto itself, advancing a belief that it is something one can design, control, plan, or structure into an organisation. It follows that workers in an organisation with more possibilities for decision making, involvement, and co-determination will be seen as having more autonomy than workers in an organisation in which decisions are centralised (Kubicek et al., 2017). The quantification of autonomy as a commodity which can be handed down hierarchically, and which a worker possesses more or less of, unveils, however, a superficial and incomplete understanding – not only of autonomy as a concept, but of how autonomy can be built, expanded, and utilised in practical organisational settings. The concept of job autonomy, for instance, which depicts degrees of freedom from parameters set by a work description, says little about how to use and develop autonomy for organisational change and development – it merely reflects some distribution activity of management control.

A shift from a negative to a positive lens denotes autonomy at the workplace as the freedom to learn from experience and develop professional practice. Such a process of practical learning and growth may ultimately make the practitioner less reliant on organisational rules and methods, whilst promoting/facilitating the development of professional judgement (Dreyfus and Dreyfus, 1980).

Positive freedom also allows for the unpacking of “choice,” challenging its position as a key denominator of autonomy. Choice as an exercise of picking or calculating the best option is arguably a superficial and technical activity compared with the creative sensemaking involved in defining the problem and producing the alternatives to choose from Schön (1987) and Ciborra (1999). Whereas creativity knows few boundaries, choice-making is deemed dependent on those parameters already defined by open-ended creativity. It follows that choice is a poor and marginal denominator to measure the quality, not to say quantity, of autonomy. The choice is a technical-rational caricature of creativity, and closer to negative freedom than positive (Dehlin, 2020). Understanding autonomy at work is to understand the workings of the creative mind; acknowledging both how it sets itself apart from, and (simultaneously) is part of, the organisational milieu.

### Autonomy at Work and Rules

The empirical basis of our manuscript consists of two high-risk organisations. High-risk organisations are embedded with rules, yet, the question of being compliant or not is highly practical and situational (Antonsen et al., 2017). Autonomy is by some thought to be a starting point or enabler of a loose relationship with rules (Grøtan, 2015), which may promote intentional rule-breaking. In the following, we will pursue the interaction of workers with rules as an entrance to a more practical perspective on autonomy. Research has formerly associated this with the ability to break rules when needed. Warren (2003), for instance, claims there are essentially two kinds of rule-breaking – positive and negative – and that autonomy plays a role in both. He further claims that there is theoretical support for a relation between autonomy and both positive and negative forms of deviance. Morrison (2006) suggests that workers who feel higher levels of job autonomy have a higher likelihood of rule violation, but also are pro-social rule-breaking, a concept which entails intentional rule-breaking rooted in a desire to promote the welfare of the organisation or one of its stakeholders.

The locus of autonomy in individual agency, rather than in a social dialectic, aligns with a static and homogenous view of organisations; unsuited to understand and further develop heterogeneous organisations in continual becoming (Carlsen, 2005). In the following, we seek to sketch out an interactionist perspective on autonomy.

“…by treating autonomy as a dynamic process of interaction……we can more clearly identify the positive and negative effects…” Trevelyan (2001, 496)

In the context of the workplace, it is interesting to contrast autonomy with organisational rules. In Peirce’s (1974a,b) pragmatism a rule represents a sign or symbol, mediating the relationship between “the other(s)” and “the individual.” This mediation is, as explained, the precursor for autonomy and, as a consequence, rules stand as part of autonomy, not apart from it. Ostensibly, rules depict the language games (Wittgenstein, 1997) we come to see as “organisations” (Feldman and Pentland, 2005). When rules are acted on and performed, however, they become templates for practice (Weick, 2002), which implies that, performatively, rules are always different from their ostensive character. The more turbulent, shifting, unpredictable, and nuanced the organisational reality becomes, the less explanatory and normative power rules have. Rules and autonomy form a symbiotic (not an antithetical) relationship in that the autonomy of a practitioner is measured from the level of understanding demonstrated in translating the ostensive character of a rule into performative quality (Schön, 1983; Dreyfus, 2004). Autonomy can thus be defined as the ability to breathe life into rules, spontaneously and with sensitivity to context. Autonomy is both action and reaction to how it should and can be tweaked, broken, or sometimes followed up in the details.

A rule is a boundary object that “sits in the middle,” connecting actors across boundaries. It enables relationships and meaning construction through communication (Heldal, 2008). The role of boundary objects has been captured by Carlile and Rebentisch (2003). A process view on boundary objects requires the collaborative parties (and/or individuals) to “jointly transform their knowledge” (Carlile, 2002, 452) to work successfully. This involves being able to negotiate, modify or change the particular boundary object. As such, the extent to which a rule is meaningful depends on the intersubjective judgement of organisational practitioners.

### Autonomy From a Pragmatic Lens

As opposed to Trevelyan (2001) who views that workers are “recipients of autonomy,” and that leadership is a paradoxical balancing act between freedom and control, a dialogical, pragmatist frame (i.e., Mead, 1934) allows for a view on control as a measure of freedom, and autonomy as an emerging upshot of social interaction: workers do not receive autonomy, but develop it in interactive, dialectic processes. Such interaction does not, contrary to the perspective of Trevelyan, take place between individuals seen as entities, but between (an always emerging) present and a past.

Inspiration for defining autonomy can be found in the fundamental idea of Mead that the self is a substance-in-the-becoming, not an entity unto itself (Mead, 1934). It follows that autonomy dwells in the forms and modalities through which the self develops practical judgment and actionability: Autonomy is, at its core, an activity of dealing with reality, in a reflexive, creative manner, reflexive to experience *and* the social/physical world. Dewey (1938) offers a similar explanation, saying:

“There is no intellectual growth without some reconstruction, some **remaking**, of impulses and desires in the form in which they first show themselves” (64).

Autonomy involves *interacting with* the social and physical context and, as much as autonomous action is never free from external imposition, it is at its core characterised by an open-ended, innovative forming of meaning, not an escape from external control. Autonomy is not found in an either/or logic between the individual and “the other” (e.g., a colleague, an organisation), nor between positive or negative freedom, but in both, and in logic (Stacey et al., 2000) by which the individual sees himself as both inevitably connected to all others (negatively), and also as uniquely different. In the words of Mead (1934):

“Consciousness as such refers to both the organism and its environment and cannot be located simply in either” (332).

Uniqueness implies the difference between someone and something; it emerges from contrast. The baby infant, Mead explains, must first take the role of his mother to invent himself as different. His uniqueness presumes kinship, and by consequence, autonomy resides in the reciprocal combination of negative and positive freedom. Any action becomes a reaction, spurring further reaction, and to the extent something genuine and unique emerges, something *autonomous*, it is nonetheless social.

### Summing Up the Theory: Autonomy as Rule Dynamic

We have traced a tendency in previous research to oversimplify autonomy, portraying it as some condition, ability, or possession binary to physical and organisational context, rules being an essential part of this. While autonomy is regularly presented as a signifier of the extent to which practitioners are successful in freeing themselves from external constraints (negative freedom), we have argued that a positive freedom perspective allows for a richer and deeper understanding. Collapsing negative freedom into positive freedom implies that autonomy in the workplace is essentially a process of open-ended creation and maturation sensitive to, and dependent on, organisational context. Autonomy in the workplace involves developing a personal voice, on which it is possible to build professional expertise and judgment. Without autonomy, the greatest skills can amount to little more than mindless routinisation and imitation (Dehlin, 2020), but by learning from experience and reacting to contextual cues (Weick, 2002) skills may be utilised and transformed into autonomous (expert) action. Autonomy is not developed in isolation from the organisational context but as part of it.

Concerning the relationship between autonomy and rules, we have used process thinking to advance an understanding of autonomy as a (pro)active relationship with rules, attentive to organisational aims, intentions, and context. Autonomy and organisational rules are mutually constitutive in practice. Seen as boundary objects, rules are linguistic externalisations of meaning, always sensitive to change and context, and thus, rule compliance or rule-breaking are mere surface aspects of a deeper organisational activity, or dance” in which sense and rationality are continually contested, negotiated, and altered in the pursuit of pragmatic functionality, to come up with actions and solutions which, from a given perspective, are perceived to be practically workable, sound, and sensible.

**FIGURE 1:**
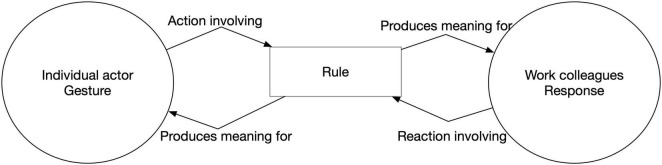
Autonomy as a rule dynamic.

Figure 1 illustrates autonomy as sensemaking, utilising rules as significant symbols (Mead, 1934), as linguistic tools (Peirce, 1974a,b), in an emerging social context. Here, any action (gesture) spurs a reaction (response), and it is in the reaction that the actor understands the social, intersubjective meaning of the gesture (Mead, 1934; Weick, 1995). It follows that positive, autonomous action is by default spontaneous but also sensitive to context response (as in negative freedom). Metaphorically, this dynamic resembles a kind of “dance,” by which sensemaking emerges continuously and jointly around emerging interpretations of the rule.

## Materials and Methods

To investigate autonomy as a practical phenomenon in a high-risk organisation we draw upon two case studies that are now presented.

### Why a Two-Case Study?

Previous research argues that autonomy may mean qualitatively different things in highly flexible work settings characterised by indirect control (i.e., “professional organisation”) than in work settings that are highly regulated and externally controlled (i.e., “machine bureaucracy”). From the perspective of previous research, autonomy for a health professional at a hospital would be expected to be different from that for an automation worker at an industrial plant. An empirical study of a high-risk hospital unit and a high-risk factory, we depict, would put these assumptions to the test, and provide valuable data on the dynamic of autonomy and rules at work and, to the extent patterns are found across the two datasets, it would allow for greater generalisation of findings and implications. Further, while less push/pressure from rule (based) restrictions may be generally beneficial in highly regulated and externally controlled work settings, it may be accompanied by greater uncertainty and ambiguity [see e.g., Johlke and Iyer (2013)] in highly flexible and indirectly controlled work settings. A process view may deepen our understanding of the complexity and nuances permeating the dynamics of autonomy and rules at work and, by proxy, provide guidance to appropriate management practice.

### Case 1: Case Description High-Risk Plant

Case 1 is a high-risk plant consisting of approximately 400 workers. Our main area of interest is the electrolysis department, with nearly 120 operators. The department is divided into operator teams, working on shifts. The teams include a shift manager, a role that is designed as an appendage to being a normal operator. The shifts are overseen by area managers, who serve in an administrative, managerial role. The general responsibility of the production teams is to monitor the production process. This consists of monitoring key parameters of the temperature and mixture of the cryolite bath, venting out fluorised carbon gases by inserting wooden rods into the gas bubbles in the bath, and repositioning and changing the anode rods when needed. Occasionally, the inside of the ovens needs to be cleaned by removing solidified aluminium, which attaches to the walls of the oven during electrolysis. The operational structure is planned and organised in accordance with the lean management philosophy, singling out and emphasising activities contributing to the value chain. This adds up to a work situation for the members of the production teams characterised by a high level of routine tasks performed in predictable cycles, but which are combined with a low tolerance for operator errors (narrow safety margins). The plant is organised as a traditional machine bureaucracy, although with some elements of team organising.

Our main data stems from interviews and ethnographic studies.

### Case 2: A High-Risk Hospital Unit

The hospital consists of approximately 10,000 workers. It is a modernised hospital organised and governed according to the New Public Management logic. It is driven according to a managerial logic (Heldal, 2015), and has, like the industrial plant, implemented lean procedures. This implies an attention to indicators and follow-up on standards. It has a stated focus on patient safety, which implies that, in addition to lean standards and regulations, there are several safety rules to comply with. In particular, we followed and interviewed the emergency centre at the hospital. This involves both patients with life-threatening wounds and urgent treatment, as well as planned and elective procedures. Emergent services are offered by doctors and nurses on “specific” duty, but as it is impossible to plan these services, selected procedures and personnel not on duty can be rushed into prioritised tasks. The hospital is organised as a professional bureaucracy, with professional autonomy as an important asset – and unlike the plant, the working days of the hospital are impossible to fully plan.

In both case organisations, the indications are that workers are subjected to several formally stated rules, but a large portion of the work-life seemed to exist outside of, or peripheral to these rules. In total, 52 in-depth interviews were conducted (30 at the hospital and 22 at the plant). Both managers and workers (doctors, nurses, operators, area managers) were interviewed, as well as relevant quality managers and administrators.

### Data Collection

The interviews lasted from 1 to 1.5 h. The interviews at the hospital were tape-recorded and transcribed. The interviews at the plant were not recorded due to technical challenges in blocking loud noises from the machinery. However, lengthy notes were transcribed by a second interviewer to obtain as much specific data as possible. The notes were immediately coded and analysed. The interviews were conducted in a semi-structured way. The objective of the interviews, as described by Kvale (1996) was to obtain a phenomenological understanding of the thoughts of the interviewees on the subject, thereby drawing on the interpretative traditions within qualitative research (Broom, 2005). The interviewees were asked questions on their relationships with their leaders, their relationship with general rules and regulations, and their co-workers.

In addition, observations were conducted. At the plant, a total of 10 h participant observational sessions were conducted by two researchers. Operators, team leaders, and general managers were followed. The observations were not designed to be an assessment in themselves. They were also seen as a step towards understanding the work of the production teams, gaining the trust of informants, and increasing our ability to ask meaningful questions in interviews. We observed operators and their team leaders in important contexts where rules were performed: on the shop floor, during pre-briefs and de-briefs, and in regular meetings and informal meetings. Notes were taken *in situ* and then coded and analysed.

### Measures and Analysis

In particular, we wanted to investigate cases in which workers were subjected to severe restrictions and control. The main limit of restriction that we paid attention to was that of rules and how they influenced daily operations for workers/professionals. Both the interviews and the observational studies focused on the adoption of the workers and their use of rules as a practical factor in deciding how to perform daily operations as effectively as possible. Both observations and interviews corroborated important patterns in the data, which the authors deemed as a sign of saturation.

### Data Analysis

The data were analysed following what Kvale calls “*ad hoc* meaning generation” (Kvale, 1997). This entails analysing the texts in various ways, instead of following pre-decided and common routines. The analysis was conducted with both authors first reading the transcripts and notes from the interviews, as well as the notes from the observational studies. We further employed what Kvale calls “meaning condensation” (Kvale, 1996). This involves compressing long statements into shorter statements, preserving the original essence. Categories were then developed, initially by each author alone and then refined by the two authors in joint sessions. This formed the basis for a tree structure and the generation of hypotheses to guide further analysis of the texts.

We built on the framework presented by Korstjens and Moser (2018). To ensure transferability, we sought to provide thick descriptions in the presentation of the data – describing not only behaviours and experiences but also contexts. The credibility of our findings was supported by using different data sources. It was also supported by the authors being in the field over a prolonged period as participants in other projects. To comply with dependability and conformability, we strove to provide transparent descriptions of the research steps (presented earlier).

Although placed firmly in the qualitative methodological tradition where subjectivity of the researcher is recognized (Ratner, 2002), we have taken further steps to enhance objectivity in our analysis of the data *via* constant comparative analysis (Gabrielsen, 2018). Acknowledging a triangulation of theory, observations, and interviews where the researchers perspectives are seen not as objective data but one of several perspectives (Tjora, 2018), we performed several iterations between the main and co-author to develop and narrow down codes from the text transcripts. The initial coding resulted in a large number of codes, which was narrowed down through a focused coding to find the most frequent and central codes. This enhanced the analysis through selection and focus (Gabrielsen, 2018), which enables more analytical control (Silverman, 2001).

## Empirical Data

We will, in the following, present data from the two cases, with specific attention to choose situations in which rules played a significant role in constricting viable paths of action. Particularly we address issues concerning autonomy and practical judgement as related to the organisational context (situation), problem-solving, and the role of a professional community (of colleagues).

### The Mechanical Bureaucracy: The Plant

At the plant, the shop floor was filled with heavy vehicles in motion. In addition, pedestrians were working with different ovens going back and forth. The shop floor was crowded, much like the roads and sidewalks of a big city. The floor had its own “traffic” rules with which both drivers and pedestrians had to comply. And, for most of the time, this had a coordinating effect on the cooperation at the floor which, allegedly, made it safer. Pedestrians were to walk within the confines of a yellow safety line, keeping away from backing vehicles, so that drivers could maintain focus on other things. A challenge was, however, that the plant also had developed rules for efficient performance. These rules were developed in accordance with the “lean” philosophy, with clear attention to time standards and reduction of waste. Using excess time or prolonging distances was regarded as waste. In effect, efficiency rules in practice overrode the safety rules. For instance, workers were cutting corners on the shop floor, crossing yellow lines that should not be crossed to arrive in time. Further, workers riding bikes were causing accidents as they collided with heavy equipment, not complying with rules of keeping within the same yellow lines to maintain traffic coordination. The workers intended to be efficient. The rules of efficiency were thus at odds with the rules of safety. The shop floor manager even stated that “…*if we were as good at following the efficiency rules with safety, we would be much safer*…*”.* Still, efficiency indicators were the primary tool of management of the manager, as these numbers were often replicated on a board called the “Lean board,” which was updated daily and continually used as a feedback tool. Although the yellow-line crossings occurred frequently, they were only given attention when accidents happened.

Efficiency was largely associated with positive feedback from managers, while safety was associated with more authoritarian and disciplinary feedback. One of the managers was observed when he reprimanded one of the workers for inappropriately using the bikes. Normally a sociable and quiet manager, he now turned quite hash and strict – like a parent disapproving the actions of his child. In contrast, efficiency was approached by managers in a more motivating fashion – seeking to inspire workers to achieve indicator goals.

At the plant, there were also power relationships related to rule-breaking, however, more hidden. The plant had, over several years, gone through reorganisation. Previously, the shop floor was run by a foreman who had responsibility for all workers. The shop floor was now organised into teams, with team managers. The old foreman was still working there but was not in a management position. Still, he was the one really governing the shop floor: Based on the interviews, it was revealed that decisions always had to include Rory (nick). Decisions that surpassed him, or failed to involve him, tended not to be carried out. It was all a matter of power relationships that did not appear in the formal hierarchy. There were no reported incidents of rule-breaking with this as a factor, but the team leaders complained that the workers would comply with these power relationships rather than order from them. When orders and social hierarchy were coordinated, it was OK, but there was a real suspicion by managers that this could lead to rule-breaking.

The workers at the plant also recognised that there were too many rules. Their approach to this was to comply with the rules that were important for them to follow for pragmatic reasons. They would typically only follow two or three of the key performance indicators and they viewed the rest as simply “nice to know.” One of the group leaders stated that

“…you soon learn to know which indicators to follow and which indicators you can abide…”.

The choice of which rules to follow was according to one of the team leaders, those judged as relevant for the production process. For instance, the temperature of the ovens was one of the most important indicators, because when too low it would indicate no production and too high a risk for explosion. Many other indicators were measured and put on the key performance indicator (KPI) board on the wall, but with little impact on the actual process, they were ignored. These represented rules, then, which were neither complied with nor broken. They were simply irrelevant. A different situation would occur if any threshold was broken - then the workers would need to pay attention to this indicator.

As described earlier, there was a clear social hierarchy at the plant reflecting both old times and the present local community. This organic social organisation outweighed the formal rule hierarchy. The shop floor was governed by rules that nobody had written down and which were seldom expressed explicitly. All the same, without knowledge of these rules, you would risk breaking the social system. For instance, a formal routine was implemented mandating a cross-shift meeting at the ending of one shift and the start of another. Such a meeting never took place, however, as the workers preferred a more informal chat. Several other formal routines were also superseded by informal structures. These were not considered aberrations, because they were not grave enough to process through the system. However, going against them ran the risk of quite severe retributions, for instance, not being invited to the local party in the afternoon. Another interesting characteristic of the plant was that the life outside the plant – the community of the small town - governed life inside the plant. The formal rule system was never as important to the workers as the informal network based on social relationships built in the community of the small town. Team managers would, for instance, hesitate to reprimand a fellow co-worker for rule-breaking if they were going to a family barbecue with them in the evening. The community network was reproduced within the plant. For example, one of the team leaders expressed reluctance to perform his duties, sometimes involving reprimanding, as he would meet the same workers in other settings outside the plant.

### The Professional Bureaucracy and Autonomy: The Hospital Unit

The emergency centre at the hospital experienced similar issues with conflicting demands. Nurses were required to follow patients whilst doing magnetic resonance (MR) scanning, a procedure done by radiologists in a different building than where nurses would normally reside. A problem was that the patients were often left in the corridor as the nurses could not wait for them to be “handed” over. The radiologists for their part also lacked time to meet up with the nurses. As a consequence, patients were often left alone to wait, and the radiologists were blamed for their bad experiences, and ultimately, reported the nurses for rule-breaking. Even if there was a rule stating that patients should be liaised by health personnel all the way, the nurses were simultaneously required to perform other tasks in their home department, which required them to head back as soon as possible. The deviation from the liaising rule, if reported, was not evidenced with their home department until a monthly safety and quality review – if evidenced at all. At the radiology department, however, these deviations were always reported and high on the “to-do list” of the quality officer. As there was little cross-sectional communication aside from casually meeting each other at the hand-off, the deviations were not prioritised with the nurses because they regarded them as a radiologist issue (they had more important tasks to attend to).

The centre was governed by a coordinator (nurse), who was responsible for prioritising patients, booking operating rooms, and administration in general. On manuscript, this role resembled something close to a technical operator, responsible for keeping the machinery going. This was also how she was trained. In reality, however, she carried out a tough and demanding leadership role. For instance, she had to navigate in, and between, two different organisational cultures of surgery, that of soft tissue surgery and orthopaedics. Where surgeons were normally accustomed to emergent procedures, orthopaedists were used to preplanned procedures. This led to significant differences in how the two professional groups related to a rule set, as particularly evident in a doctrine developed for the purpose of prioritising patients on the basis of degrees of emergency: red (procedure within 8 h), yellow (procedure within 24 h), and green (procedure within 72 h.) Even though this was developed jointly by the two professions, the system seemed to take a life of its own. Doctors shifted the colours as they saw fit; for instance, changing from yellow to red if they wanted to perform surgery before ending the shift or the other way around – the next morning. As the professions used the same operating rooms (and anaesthetic personnel), this led to ongoing tensions and conflicts. One doctor exclaimed that “…this system functions only with a lot of dialogue…” but seemingly, as they did not understand the *modus operandi* of each other (for instance, orthopaedists complained that for surgeons, all patients were Red), the dialogue between the two groups had to go through the coordinator. The coordinating nurse thus had to use a lot of practical and social judgment in navigating the logistics between the groups, whilst at the same time, all parties agreed to the importance of following the rules, to begin with. One anaesthetist observing this intriguing social practice from the outside stated that each doctor seemed to be “guarding” his/her own patients, a type of sub-optimisation, as it were, where group identity came before patient concerns of the other group.

The hospital contained, in general, myriad unwritten rules, that evidenced themselves in the varying levels of *compliance* with written rules. This was, for instance, evident in their usage of the formal reporting system, because what was reported was sometimes governed, or at least influenced, by other things than what they were intended to. First, the system was managed by nurses, as doctors would guard their patient’s time and not do administrative work. Consequently, doctors were reluctant to report errors and mistakes to a nursing-administered system. Errors made by doctors were not supposed to be noticed by nurses. Second, working with quality and patient safety was typically confused with what could technically be reported into and captured by the system, with the result that many complexes, tacit, and professional issues were concealed or officially left unaddressed. Cancer nurses were, for instance, unduly preoccupied with patient falls, seemingly with little other reason than ease of reporting it and thus bringing it to the attention of managers. Doctors resolved this issue by separating “quality” (issues that could be quantified and reported) from “medical quality” (issues that concerned strictly professional matters). Third, the system was used to distribute blame. For instance, during summer vacation, the system came into use significantly more than was usual. During the summer, the interns were dissatisfied with the substitutes and thus reported them in the system. Similarly, and on a more general note, people in the conflict used the system to harass each other through reporting deviations using a name, blame, shame approach.

The complexity of the hospital, with links and relationships between professions and centres, caused more problems. The emergency centre was reliant on proficient pictures of patients, but could not always be trusted with this. There were several mishaps at the unit for picture diagnostics that were easily explainable but hard to fix. Radio-graphologists had the task to x-ray patients for a number of reasons, and sometimes this involved cancer patients and the task to take pictures of a cancer lump inside the patient; oftentimes before going into a surgical procedure at the emergency centre. This entailed, in many instances, inserting a liquid that would illuminate blood vessels. The challenge was that this liquid would interact with cytostatika within a certain time amount - and it would lead to a mishap if it was inserted too soon after the cytostatika treatment. The problem was that radio-graphologists would not automatically get information on the possible cytostatika treatment from the doctors, because they were not allowed insight into the journal of the patient. Patient laws prevented other personnel other than the treating doctors from having this kind of information. When patients were conscious, this would not be a problem, because they could then tell the radio graphologists when they last had been treated with cytostatika. A different situation occurred when the patients were unconscious. In this case, the graphologists had no other option to obtain the information than to investigate the journal of the patients (or ask the doctors, but they were rarely available when needed). The interesting matter here is that, in order to avoid patient harm, the radio graphologist would have to break the rule. Many graphologists hesitated to break this rule, not only because it was a formal rule but also because it would entail a responsibility that they did not have as a radiograph. It violated the professional hierarchy. Others reported however that they would sometimes break the rule in the interest of the well-being of the patient. What is important for the graphologists in this regard was not so much the rule-breaking *per se*, but the responsible doctor. They knew that some would applaud such a pro-active behaviour, albeit breaking a rule; while others would frown upon it.

There were further reported deviations on mishaps due to the health professionals being stressed and having little time. They were plagued with more and more tasks to be done within the same amount of time, due to economic cut-downs and efficiency claims. This led to higher levels of stress, which in itself was a cause of deviations. For instance, the regulations clearly stated that workers should have time for lunch, and not be on duty for too many hours in a row. These regulations were now often overridden to meet efficiency numbers. However, they had no time to log on to the system to report these kinds of deviations. The system itself was complicit in concealing what it should report. The numbers showed a decline in deviations due to stress when the reality was the opposite. One of the quality managers acknowledged that if workers were to report every broken rule, they would have no time left to treat patients. So, there was a silent agreement that the rules could be overridden, simply because there were too many and it was not possible to comply with them all at once. Only the rules that were highlighted by the management as important to follow were attended to. Rule-breaking that was irrelevant to the managerial agenda was either not given attention or not reported at all. They resided outside of the formal system. The breaking of rules that were on the managerial agenda, on the other hand, were responded to and, even sometimes used in a way that was beneficent (for instance to promote learning).

## Discussion

How is autonomy developed and practiced in a high-risk organisation? Morrison (2006) suggests that pro-social rule-breaking is driven by other factors (job cognition and situational variables) rather than dispositions, which we argue is evident in the similarities between our two cases studies despite differences in organisational structures. More than structure, we suggest that autonomy is a matter of dynamic interaction with rules in a social context – dancing with rules. We will present four different modalities of autonomy and rule dynamics based on process thinking, that illustrate how actors interpret and use rules in a social context. Based on the data presented, we developed a taxonomy depicting these four modalities of autonomy-rule dynamics. The taxonomy builds upon Meads’s concept of “gesture” and “response,” which we translate to “action” and “reaction” - a dialogical sense-making process (Weick, 1995) involving the rule as a boundary object, as presented in the theory section. From our data, we see that this dialogue differs with regards to either a weak or strong “negative freedom” action or a weak or strong “positive freedom” reaction.

### Autonomy-Rule Dynamic as Passivity and Resignation

This is the case in which *positive freedom* is rarely seen and rule-based actions are minimally spontaneous or pro-active. *Negative freedom* is neither pronounced in the responses towards these actions, meaning that the rules are, to a small degree, imposed or justified. The practical equivalent here is that of actors resigning into passivity, making choices but without conscious attention to neither personal (professional) intentions nor others. The professionals described kind bewilderment with regard to an abundance of rules, where arbitrariness could influence which rules one complies with. A similar situation took place at the plant when the plant manager expressed regret in failing to be as good with safety rules as with efficiency rules. Knowledge and attention towards the issue were thoroughly demonstrated, but the action was overtaken by resignation. This all entails a kind of induced passivity in choice-making and responding to choice-making; entailing a low degree of personal intention (*positive freedom*) but also little attention from the receiving others, for instance, the manager (*negative freedom*). As a choice situation, we suggest calling such practicing of autonomy resignation, a rule relationship based on passivity to either task or others (McGrath, 1991; Rodríguez-Sánchez et al., 2017), where the meaning of the rule is nearly made irrelevant, dialogue is weak, and autonomous expression and action is weak.

### Autonomy-Rule Dynamic as Loyalty and Conformity

This is the case in which *negative freedom* is very pronounced while *positive freedom* is less spontaneous and pro-active. In other words, rules are imposed from a hierarchical position, intending lesser degrees of freedom for the individual actor. This was evidenced in both cases with the workers and professionals choosing to abide by either management positions or off-plant hierarchy. For instance, with the barbecue situation, rule-breaking was socially expected and thus imposed on the team leaders. The off-plant society was, in this regard, more imposing than formal rules at the plant. Radio-graphologists complied with the rule of entering the patient journal if specific persons were on duty. This situation may be understood as actors responding out of loyalty, not to the rule but to the people at the receiving end. Both examples are matters of conformity and acceptance. In the latter case it may be understood as being loyal to the task (San Martín-Rodríguez et al., 2005), and in the former as a case of being loyal to the group (McGrath et al., 2000; Rodríguez-Sánchez et al., 2017). Both involve a surrender of individual will. We describe this as a relationship based on conformity and adherence, which may also be associated with fear of condemnation (Pescosolido, 2003). The dialogue here is imbalanced, inhibiting the constructive feedback that is vital for learning (Argyris and Schön, 1996). The rule becomes a boundary object where its meaning is governed by higher levels of the hierarchy with the intention to constrict (subordinate) behaviour.

### Autonomy as Self-Promotion

This is a case in which positive freedom is prominent, largely at the expense of negative freedom. Self-promotion is related to self-governance, independence, and sovereignty (Warren, 2003), and can be understood as individuals acting freely and intentionally, with greater attention to personal wellbeing than to that of others. Here self-interest trumps empathy. A practical example from the dataset concerns the health professionals making reports for the sole purpose of inflicting damage on others. For instance, doctors were observed to report other doctors with whom they were in conflict; whereas nurses reported the newly arrived interns, to state an example. In this regard, autonomous acts of self-promotion may be understood as what Vardi and Weitz (2004) have coined “organisational misbehaviour,” intentional acts that violate core organisational and/or societal norms. In this regard, autonomous acts of self-promotion may be understood as acts of power, exemplified also by the informal foreman of the plant resembling an obligatory passage point (all decisions had to go through him) (Callon, 1986; Latour, 2005). Examples also include the manner in which surgeons and orthopaedists argued over priorities or degrees of emergency, prompting a sub-optimal distribution of patients. As these actions demonstrate self-promotion, acting predominantly out of self-interest, they signify a lack of context-sensitive professional judgement as to how rules should be made to work to provide the best possible outcome. The rule then becomes a boundary object, with the meaning managed solely by the individual. Such suboptimal behaviour is inclined to produce bizarre effects, as shown in the case of the coordinating nurse, wasting energy on negotiating sense between doctors with primadonna-like behavioural features.

### Autonomy-Rule Dynamics as Co-generative Learning

This is a case in which both positive and negative freedom are prominent, ensuring an ongoing dialogue and constructive development process. Such joint construction of meaning also implies jointly agreeing on the modification of rules when seen fit as the parties involved “jointly transform their knowledge” (Carlile, 2002, 452). This autonomy-rule dynamic then involves being able to negotiate, modify, or change the particular boundary object on pragmatic grounds and for practical purposes. A viable example concerns the graphologists going beyond the rule not to make entries in the patient journal, out of an intention of doing whatever is best for the patient (*positive freedom*), which could receive both consent and praise from some higher-level managers and doctors. Such action and reaction, involving acting outside of formal directives, may presuppose a high level of interpersonal trust between the parties, a psychological safety free of the fear that the action would be frowned upon (Edmundson, 1999), but can also be performed from a basis in idealism or simply as a matter of practical judgement. In any case, this represents an autonomous call for recognising other intentions than what is formally prescribed and, in addition, that such re-invention of intentionality is a matter of intersubjective negotiation. As a result, organisational development is achieved by way of pushing collective, intersubjective boundaries (Wiley, 1988), but not at the expense of individual concerns. On the contrary, this autonomy-rule dynamic is a process of co-generative learning (Elden and Levin, 1990), by which individual and collective learning are mutually constitutive, and where rules as boundary objects are instrumental to (and not constrictive to) open-ended creative judgement.

## Conclusion

Autonomy has been hailed as an important constituent of both job satisfaction and job performance. In this article, we pursue a practical understanding of how autonomy may be understood in day-to-day operations, and how it relates to the application of, and interaction with, organisational rules. Based on two case studies of normal-day operations in two high-risk organisations, we sketched out four different modalities of autonomy-rule dynamics, which suggests that autonomy and rules form a complex and nuanced dynamic sensitive to context and individual and organisational intention. The larger process of making sense of rules, of understanding context, intention, and what is the appropriate action, indicates that autonomy in any sort or modality is far more complex than mere decision making. Our analysis gives many examples of how autonomy is only superficially defined by making the decision, and in accordance with Ciborra (1999), we find that below the surface of any decision is a negotiation of sense, doubt, despair, priority, power, and interest, both internally and with other interested parties. If anything, our analysis induces an acknowledgement of those processes of phronesis (Dehlin, 2012) in producing the best outcome under difficult, often conflicting, conditions and attributes the significance of autonomy to that, which implies a warning against seeing autonomy merely as antithetical to external constraints. For purposes of effective organising, to the extent the organisation pursues change and readiness for change, it is practical judgement that should be developed, as both a collective and individual undertaking, not a mere (technical) adjustment of formal directives or a removal of (negative) constraints.

### Limitations

We acknowledge the limitations of using two high-risk organisations as the empirical basis, in that our argument builds on the significance of context specificity. Further, we describe normal-day operations relating to rules, which may look very different under more turbulent circumstances. Future research may look into other methodological angles and empirical contexts related to autonomy and rules; for instance, rule-breaking and creativity in innovation teams and autonomy in management positions. Another suggestion is to use our findings to develop more quantifiable methods. This could be, for instance, in the way of a development of checklists (Gawande, 2010), as demonstrated in practical use for practical organizational development by Weick and Sutcliffe (2007). It could also serve as a development of measurements scales of the different weightings of positive/negative freedom, as demonstrated in the development of the SYMLOG and systematizing the person group relationship (SPGR) tool for team development [see Osgood et al. (1957) and Sjøvold (1995)].

### Implications for Practice

Together, our four modalities portray autonomy, not as some commodity that can be possessed and manipulated, handed down the hierarchy (as a gift) or controlled by management structures, but as an emergent feature of professional practice which conveys both positive and negative aspects of freedom, often both at the same time. Further, our analysis suggests that, in organisational practice, positive and negative freedom are not antagonistic but rather, aspects of “the same,” as particularly visible when radical new sense and action are formed (i.e., co-generative learning). As a practical implication autonomy may not be structured into an organisational chart or written down in a job description. Rather, autonomy should be understood as a matter of training and development, by which rules are understood as tools of sensemaking. Our four-modality model may serve as a tool for reflection in this training, and if discussed together, may build trust and mutual understanding in the relationships between actors. Rules are part of autonomous practice, but they require context-sensitive, intelligent adaption. Our two cases point strongly to this, and a take-away is that in (every day) organisational practice it is seldom a question of following rules or breaking them, rather, the concern is how to make sense of them and produce sound action, with the intention, problem, and context considered.

## Data Availability Statement

The raw data supporting the conclusions of this article will be made available by the authors, without undue reservation.

## Ethics Statement

Ethical review and approval was not required for the study on human participants in accordance with the local legislation and institutional requirements. The patients/participants provided their written informed consent to participate in this study.

## Author Contributions

FH had been main responsible to wrote the manuscript. Both authors contributed to the article and approved the submitted version.

## Conflict of Interest

The authors declare that the research was conducted in the absence of any commercial or financial relationships that could be construed as a potential conflict of interest.

## Publisher’s Note

All claims expressed in this article are solely those of the authors and do not necessarily represent those of their affiliated organizations, or those of the publisher, the editors and the reviewers. Any product that may be evaluated in this article, or claim that may be made by its manufacturer, is not guaranteed or endorsed by the publisher.
